# Hydrogel-Based Therapy for Age-Related Macular Degeneration: Current Innovations, Impediments, and Future Perspectives

**DOI:** 10.3390/gels10030158

**Published:** 2024-02-21

**Authors:** Chengzhi Zhang, Jiale Wang, Hao Wu, Wenhui Fan, Siyu Li, Dong Wei, Zongming Song, Ye Tao

**Affiliations:** 1 Department of Ophthalmology, Henan Eye Institute, Henan Eye Hospital, Henan Provincial People’s Hospital (People’s Hospital of Zheng Zhou University), Zhengzhou 450003, China202047000423@stu.zzu.edu.cn (D.W.); 2 College of Medicine, Zhengzhou University, Zhengzhou 450001, China

**Keywords:** hydrogel, age-related macular degeneration, anti-VEGF, tissue engineering

## Abstract

Age-related macular degeneration (AMD) is an ocular disease that leads to progressive photoreceptor death and visual impairment. Currently, the most common therapeutic strategy is to deliver anti-vascular endothelial growth factor (anti-VEGF) agents into the eyes of patients with wet AMD. However, this treatment method requires repeated injections, which potentially results in surgical complications and unwanted side effects for patients. An effective therapeutic approach for dry AMD also remains elusive. Therefore, there is a surge of enthusiasm for the developing the biodegradable drug delivery systems with sustained release capability and develop a promising therapeutic strategy. Notably, the strides made in hydrogels which possess intricate three-dimensional polymer networks have profoundly facilitated the treatments of AMD. Researchers have established diverse hydrogel-based delivery systems with marvelous biocompatibility and efficacy. Advantageously, these hydrogel-based transplantation therapies provide promising opportunities for vision restoration. Herein, we provide an overview of the properties and potential of hydrogels for ocular delivery. We introduce recent advances in the utilization of hydrogels for the delivery of anti-VEGF and in cell implantation. Further refinements of these findings would lay the basis for developing more rational and curative therapies for AMD.

## 1. Introduction

Age-related macular degeneration (AMD) is a multifactorial neurodegenerative condition of the macula that leads to irreversible visual impairments. As a worldwide healthy problem, it presents a public concern which incurs a substantial economic burden with fast population growth [1]. In its early stage, AMD is characterized by the formation of drusen, the focal accumulation of extracellular deposits, including lipofuscin, cholesterol, lipids, and immune system-associated elements [2]. If the drusen become enlarged and deteriorated, it will lead to the detachment of the retinal pigment epithelium (RPE). Clinically, AMD can be classified into wet and dry forms in the advanced stage. For dry AMD, the presence of sharply demarcated atrophic lesions in the outer retinal layer stems from the loss of the RPE, photoreceptors, and the underlying choriocapillaris. As these retinal lesions occur and expand, they precipitate irreversible deterioration of visual function. For wet AMD, the fragile neovasculature from the choroid breaks into the Bruch membrane and forms a choroidal neovascular membrane. The process of neovascularization will destroy the architecture of the overlying RPE and the outer retina, thereby causing serious visual impairments [3]. Although wet AMD only accounts for 20% of patients with AMD, 90% of these patients have severe visual impairment [4]. Currently, there is no effective treatment can halt the pathological progression of dry AMD, despite its ever-increasing prevalence. Anti-vascular endothelial growth factor (anti-VEGF) agents represent the most common treatment for alleviating wet AMD. The currently available anti-VEGF compounds, with half-lives typically lasting 5 to 8 days, exhibit limited effectiveness in the eye’s posterior segment [5]. The hinderance of anti-VEGF optimal efficacy can be attributed to its low bioavailability and high clearance rate. In order to ensure the optimal therapeutic outcome, multiple intravitreal injections of anti-VEGF drugs are adopted in clinical practice. However, repeated intraocular injection may induce various complications such as endophthalmitis, intraocular inflammation, intraocular pressure elevation, and rhegmatogenous retinal detachment [6]. Moreover, the invasive procedures will increase the physical suffering of patients and reduce their compliance.

Hydrogels are known for their remarkable ability to retain a large amount of water within their intricate three-dimensional polymer networks [7]. They serve as a platform to refine existing anti-angiogenesis agents and hold the potential to evolve into an effective treatment modality. The perfect biocompatibility of hydrogels makes them readily injected into eyeball as a drug delivery system or a tissue engineering scaffold. Water-soluble drugs can be encapsulated in the pores formed by swelling in water and interacting with water molecules, thereby avoiding interference from the environment [8]. In addition, the desired release profile of loaded drugs can be achieved by adjusting the pore size, polymeric materials, and specific physical or chemical stimuli [9]. Moreover, the microenvironment formed by hydrogels is able to preserve the biocompatibility and viability of implanted cells. With a similar structure and biological properties to the natural extracellular matrix (ECM), hydrogels provide a safeguard for the growth, development, and precise functional action of cells [10]. Eventually, implanted hydrogels can be degraded by optical clearance within the vitreous and subretinal space to obviate indiscernible interference and complications. In this review, we introduce recent advancements in the design and development of hydrogel-based treatments and elucidate the main impediments during their clinical translation process. Further refinements of these findings would enrich our knowledge of hydrogels and pave the way for developing curative biomaterials in clinical practice. We believe that, with persistent scientific and technical breakthroughs, the utilization of hydrogels in AMD treatments could herald a new era in ophthalmology.

## 2. Pathology of AMD

Oxidative stress has been identified as a crucial pathogenesis factor that contributes to the occurrence and development of AMD. Reactive oxygen species (ROS) generated by lipid peroxidation of the RPE, photosensitizer molecules, intense light exposure, and high oxygen levels within the macular region establish a milieu that fosters cellular oxidative stress. The retina, particularly in the macula, exhibits intense blood flow and high oxygen consumption due to its vigorous metabolism, resulting in abundant ROS generation, even under physiologic conditions. In particular, pathological stimulus can destroy the balance of the mitochondrial electron transport chain and give rise to the overproduction of ROS. In response to oxidative stress, intracellular antioxidant genes demonstrate augmented transcriptional activity and bolster the antioxidant defense system. Nevertheless, this antioxidant response becomes exhausted due to persistent oxidative stress [11]. Mitochondrial DNA within RPE cells is susceptible to oxidative damage. The mitochondrial genome represents a main target for the oxidative stimulus. These cytotoxic ROS disrupt mitochondrial DNA integrity and attack the proteins and lipids within the mitochondrial membrane. This disturbance leads to the opening of mitochondrial permeability transition pores followed by the release of cytochrome C along with other apoptosis-inducing factors into the cytoplasmic compartment [12]. Researchers have demonstrated that hydrogen peroxide-induced oxidative damage can inhibit the expression of the anti-apoptotic protein Bcl-2 and enhance the expression of the pro-apoptotic protein BAX within the mitochondria of the RPE, thereby activating the apoptosis signaling pathway [13]. Extensive RPE damage critically undermines retinal homeostasis, interrupting the transport of nutrients to photoreceptors and the clearance of their metabolism wastes, ultimately leading to photoreceptor death. These pathological alterations disrupt the transmission of visual signals, resulting in retinal dysfunction.

**Figure 1:**
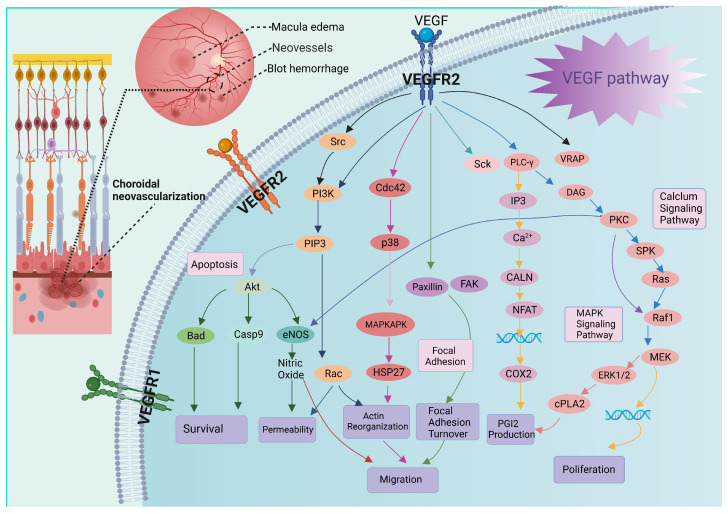
The combination of VEGF and VEGFR2 triggers the formation of new blood vessels. Created with BioRender.com, accessed on 22 January 2024.

During the pathological process of wet AMD, VEGF is secreted by RPE and subsequently stimulates the abnormal proliferation of choroidal capillaries and the formation of neovasculature with compromised integrity. The resulted plasma leakage will cause rapid vision loss within a short period of time. VEGF is a homodimeric glycoprotein with a molecular weight of 34–45 kDa [14]. Endogenous VEGF can be produced by perivascular cells in the retina and exert biological effects on endothelial cells in a paracrine manner through specific combinations with its receptors [15]. Thus far, three main VEGF receptors have been identified, including VEGFR1 (FLT-1), VEGFR2 (KDR), and VEGFR3 (FLT-4). The binding of VEGF to domains 5 and 6 on the cell membrane surface activates the tyrosine kinase activity of the receptors, leading to their dimerization and the subsequent self-phosphorylation of cytoplasmic tyrosine kinase residues via IG-like domain 4 [16]. This activation initiates the downstream signaling that is involved in the proliferation and migration of vascular endothelial cells. Proper functioning of the VEGF signaling cascade is essential for normal vascular development. However, excessive VEGF can induce aberrant angiogenesis, enhance vascular permeability, and cause tissue edema under pathological conditions. For instance, VEGF-A, the most potent angiogenic factor, can promote neovascularization, triggering a cascade of reactions closely intertwined with the fundus pathologies observed in AMD [17] (Figure 1). In clinical practice, the prevailing clinical approach for wet AMD involves the intravitreal injection of anti-VEGF agents at intervals ranging from every 1 to 3 months [18]. Nevertheless, repeated intravitreal injections can reduce the compliance of patients and increase the risk of complications. In this context, hydrogels have been used as a biological material to achieve the controlled release of anti-VEGF drugs.

## 3. Hydrogels and Ocular Delivery: Properties and Potential

Hydrogels possess three-dimensional networks which are capable of retaining significant amounts of water. These hydrophilic polymers are renowned for their marvelous multifunctionality and biocompatibility. The utilization of hydrogels extends across a broad spectrum, from drug delivery systems to tissue engineering scaffolds [8,19,20]. In ophthalmological practice, hydrogels have garnered attention for their potentials in revolutionizing the therapeutic strategy of AMD. These hydrogels can encapsulate therapeutic agents and provide sustained drug delivery at the target location while minimizing systemic exposure and the incidence of complications caused by repeated injections. In particular, recent advancements in the design and fabrication technology of hydrogels have paved the way for transforming the landscape of ocular therapeutics.

### 3.1. Classification of Hydrogels

In the multifaceted realm of polymeric technology, hydrogels are classified into natural hydrogels, synthetic hydrogels, hybrid hydrogels, and responsive hydrogels according to their source and responsiveness to environmental stimulus.

Natural hydrogels, such as collagen and hyaluronic acid, are derived directly from biological systems, and their inherent biocompatibility makes them particularly conducive to biological interactions and cellular integration. For instance, collagen hydrogels have been used to facilitate cell migration and proliferation during the process of tissue repair [21]. On the other hand, synthetic hydrogels are crafted from polymers such as poly (2-hydroxyethyl methacrylate) (pHEMA) and poly(acrylamide) (PAAm), offering a high degree of control over their physical and chemical properties. The versatility of synthetic hydrogels allows for the precise tuning of pore size and degradation rates, making them highly suitable for creating customized drug delivery platforms. pHEMA, for instance, has been utilized as a material for making soft contact lenses due to its unique transparency and satisfactory compatibility with ocular tissue [22,23]. Hybrid hydrogels represent an innovative fusion of both natural and synthetic materials, aiming to synergize the advantages of both categories. These composite materials can combine the favorable biological interactions of natural polymers with the mechanical strength of synthetic hydrogels [24]. For instance, gelatin methacryloyl (GelMA) hydrogel is a hybrid system that integrates the bioactive features of gelatin with the photopolymerizable attributes of methacrylate groups [25]. GelMA has been utilized in ocular applications for creating bioinks in the 3D bioprinting of corneal tissue constructs, offering a promising pathway for corneal repair and regeneration. In particular, researchers can tailor the hydrogel’s properties to the meet specific needs in ophthalmological practice such as bio-integration, controlled drug release, and structural support, ultimately aiming to improve the visual prognosis of the patient.

Responsive hydrogels represent an advanced category that is engineered to undergo changes in response to specific environmental triggers. According to their responsiveness, responsive hydrogels can be broadly categorized into five main types: pH-sensitive, temperature-sensitive, light-sensitive, ROS-sensitive, and multi-responsive hydrogels. The pH-sensitive hydrogels swell in response to pH changes in the gastrointestinal tract or other body environments with distinct pH levels [26]. Temperature-sensitive hydrogels, such as those made from poly(N-isopropylacrylamide), undergo a sol–gel phase transition at a specific temperature [27]. This property is advantageous, as these hydrogels can release their payload in response to the localized temperature change and realize the controlled drug release. Recently, researchers formulated a novel hydrogel using two thermosensitive ABA triblock copolymers, each containing furan or maleimide moieties, called poly (NIPAM-co-HEA/furan)-PEG6K-P(NIPAM-co-HEA/furan) (PNF)/poly (NIPAM-co-HEA/maleimide)-PEG6K-P(NIPAM-co-HEA/-maleimide) (PNM) [28]. Upon mixing aqueous PNF and PNM, an immediate sol–gel transition occurs within less than a minute at 37 °C. Additionally, in situ hydrogel formation at 37 °C is observed after injecting the formulation into a rabbit eye. Another study demonstrates that the thermoreversible gelation property, high water solubility, and transparent gel-forming capacity of Poloxamer 407 and Poloxamer 188 make them widely utilized in ocular drug delivery. Poloxamer aqueous solutions display temperature sensitivity and undergo a sol–gel transition at approximately 37 °C and a gel–sol transition around 50 °C, allowing them to form thermoreversible gels [29]. Light-sensitive hydrogels respond to specific wavelengths of light stimulus, allowing for the spatial and temporal control of drug release [30]. For instance, hydrogels containing azobenzene groups can undergo reversible structural changes upon exposure to UV or visible light [31]. ROS-sensitive hydrogels are designed to react to the presence of oxidative stress markers such as hydrogen peroxide, superoxide, and hydroxyl radicals. These hydrogels can be engineered to degrade or alter their structure in the presence of ROS, thereby initiating the release of encapsulated drugs [32]. For instance, hydrogels containing thioketal linkages are cleavable by ROS [33]. This feature is especially valuable in the treatment of AMD, as its pathological condition is characterized by elevated levels of ROS in the retinal tissue. Multi-responsive hydrogels combine the aforementioned stimuli-responsive behaviors, offering sophisticated control mechanisms for drug delivery [34]. For example, a hydrogel that responds to both pH and temperature can facilitate highly targeted drug release triggered by the unique microenvironment in tumor [35]. In the realm of AMD treatment, such smart hydrogels could revolutionize the administration of drugs, offering sustained and controlled release that could reduce the frequency of administration.

### 3.2. Mechanisms of Drug Release in Hydrogels

The encapsulation of drugs within hydrogels can protect them from enzymatic degradation, thereby maintaining therapeutic concentrations over extended periods. Hydrogels can release drugs through various mechanisms, such as diffusion, swelling-controlled release, degradation-controlled release, stimulus-responsive release, and microencapsulation. Firstly, diffusion is driven by the concentration gradient between the drug-loaded hydrogel and the surrounding medium [36]. It allows drugs to penetrate the hydrogel matrix into the surrounding environment. For instance, the controlled drug delivery mechanism in hydrogel contact lenses is intricately orchestrated, involving the gradual diffusion of water from the lens’s aqueous channels, followed by the subsequent processes of drug dissolution and diffusion [37]. Secondly, swelling-controlled release involves the absorption of water, leading to matrix expansion and the sustained release of drug molecules [38]. The release rate is regulated by the water absorption capacity and swelling properties of the hydrogels. Thirdly, degradation-controlled release relies on the breakdown of a hydrogel. The degradation rate can be adjusted by modifying the composition and cross-linking density of the hydrogel [39]. In the treatment of AMD, a biodegradable hydrogel could be engineered to degrade slowly in the macular region, thereby administering anti-angiogenic drugs in a controlled manner [40]. Another mechanism is stimulus-responsive release, where certain hydrogels can respond to specific external stimulus, as mentioned earlier [27]. For instance, light-sensitive hydrogels can be employed for ocular drug delivery, where the release of medication can be triggered by exposure of the eye to light [31]. Lastly, microencapsulation involves encapsulating drugs within microspheres or nanoparticles dispersed throughout the hydrogel matrix [41]. These microspheres enable stable drug release over time. For example, in certain anti-angiogenic ophthalmic medications, microspheres may shield the drug from burst release, facilitating sustained release [37]. In practical applications, the specific drug release mode depends on the design and composition of the hydrogel system, as well as the desired release profile for the drug.

### 3.3. Factors Influencing Drug Release in Hydrogels

Several key factors can affect drug release from hydrogels. For instance, temperature can have impacts on the swelling behavior and porosity of hydrogels. These alterations, in turn, affect the diffusion and release efficacy of drugs. At lower temperatures, the polymer coating layer is swollen, leading to a quicker drug release rate, while at higher temperatures, the release rate is slower due to the collapse of the hydrogel [42]. Hydrogels containing chitosan can retain a liquid state at room temperature and undergo gelation upon injection into the eyes [43]. Ocular biological barriers also play a crucial role in the release and absorption of drugs in the eyes. The corneal and bulbar conjunctival epithelia act as major barriers for drug absorption. With the topical administration of drug formulations, less than 5% reaches the anterior part of the eyeball, and an even smaller fraction, often less than 1%, reaches the posterior eye segment [44]. As for intravitreal administration, the vitreous humor exhibits a relatively loose structure in both its normal and liquefied states, with diffusivities that are not significantly different from those of water. Notably, the experimental diffusion data for the small molecule dexamethasone in the vitreous appeared to be higher than its theoretical diffusivity in water, implying the superiority of intravitreal administration [45]. Another influential factor is the pH of the surrounding environment. The pH level can modify the charge and swelling behavior of hydrogels, thereby triggering structural changes that subsequently influence drug release [26]. The pH of tears is around 7.4, and any variation from this pH level may result in irritation [46]. Some hydrogels are intentionally designed to respond to specific pH level, enabling targeted drug release. A study revealed that a mixture of carbopol and hydroxypropylmethylcellulose remained in a liquid state at the formulated pH of 6.0 and swiftly underwent gelation when the pH was elevated to 7.4 [47]. The presence of ions in the surrounding medium also holds significance. These ions can modify the swelling behavior and mesh size of hydrogels, thus influencing drug release through electrostatic interactions [48]. Additionally, exposure to specific wavelengths of light can induce changes in the structure or chemical properties of light-sensitive hydrogels, thereby triggering drug release [30]. These external stimuli provide a means to control and modulate drug release from the hydrogel matrices. It is important to note, however, that the specific formulation and design of the hydrogel system can also affect the process of drug release.

### 3.4. Safety of Hydrogels for Ocular Delivery

The safety of hydrogels is the foundation for their clinical application. The assurance of safety is discerned through an evaluation of potential cytotoxic effects within tissue cells, inflammatory responses, and any propensity to induce abnormal hemorrhage. In vitro, the most commonly employed method for assessing whether fabricated hydrogels are harmful to cells is through the utilization of live/dead assays. In brief, cells attached to plates are cultured with fabricated hydrogels, and their morphology is subsequently observed using a fluorescence microscope. In a recent investigation, hydrogels were assessed in relation to RPE-1 cells and acknowledged for their prevalence and sensitivity in the retina [49]. The outcomes revealed that the hydrogel, even under varying drug loading conditions, exhibited non-cytotoxicity compared to the control group. In vivo, the safety of hydrogels can be gauged through sophisticated methods such as histological analyses of ocular tissues and precise measurements of intraocular pressure [50]. Multiple studies have conclusively affirmed this throughout the study period. There were no obvious inflammatory responses or fundus abnormalities, including lesions, retinal whitening, or hemorrhage, whether in rat [37,51] or rabbit [50,52] model. Moreover, a comprehensive investigation revealed a consistent level of rabbit TNF-α in eyes subjected to hydrogel injection over the entire study period, indicating the absence of a pro-inflammatory response attributable to hydrogel injection compared with that in control eyes [49].

### 3.5. Advantages and Limitations of Hydrogel Applications

**Figure 2:**
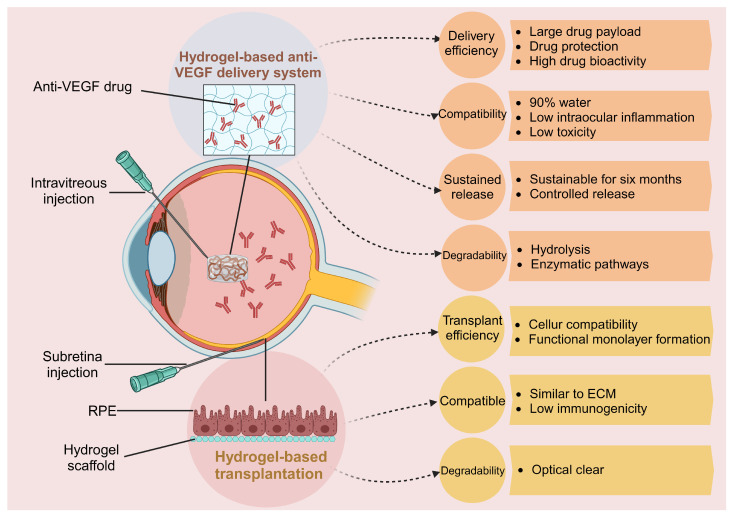
Advantages of hydrogels in ocular drug delivery and cellular transportation. Created with BioRender.com, accessed on 22 January 2024.

In the vanguard of ophthalmic therapeutics, hydrogels emerge as a superior medium for drug delivery (Figure 2). Their preeminence over other materials should be attributed to a unique confluence of properties that align seamlessly with the delicate ocular environment. The intrinsic hydrophilicity of hydrogels fosters a biocompatible interface that can reduce irritation and enhance patient comfort, a paramount consideration in eye-related applications [53,54]. The versatility of hydrogels extends to their transparency, an essential feature for any material used in the visual axis. Their clear nature does not impede light transmission, allowing for an unobtrusive treatment modality. Furthermore, the viscoelastic properties of hydrogels provide structural support to the delicate ocular tissue during movement and blinking, which is not typically afforded by more rigid delivery systems [55]. The field of hydrogel research is burgeoning with innovative prospects, as state-of-the-art advancements continue to push the boundaries of what is possible. From the integration of nanotechnology for precision release to the development of biodegradable hydrogels that eliminate the need for surgical removal after drug depletion, the horizon becomes broad and promising. In the treatment of AMD, the restoration and preservation of vision are of special importance. To this end, hydrogels may offer a beacon of hope, paving the way for therapeutics that are not only effective but also aligned with the natural physiology of the eye.

Although hydrogels offer transformative potentials for ocular drug delivery, their application is not without any challenge or limitation. One of the primary concerns is the delicate balance between hydrogel stiffness and injectability. Hydrogels must be soft enough to be minimally invasive and comfortable for the patient yet possess sufficient rigidity to maintain their structure within the dynamic ocular environment. Achieving this balance is critical, as any deviation may compromise the integrity of the hydrogel and interrupt the consistency of drug release. Additionally, the physicochemical properties that make hydrogels highly biocompatible and hydrating can also lead to rapid degradation and clearance from the eye, potentially curtailing the duration of drug release and dampening the therapeutic efficacy. To resolve this problem, the hydrogels are readministered frequently. Repeated application will increase the risk of cumulative trauma to ocular tissue and reduce the patient compliance. Moreover, the development of responsive hydrogels necessitates an intricate endeavor because the ocular milieu is less predictable than other environments. In this context, designing hydrogels that can effectively respond to ocular-specific stimuli without inducing an adverse response is a sophisticated task. In the pursuit of efficient drug delivery systems, the unwanted immune responses elicited by hydrogels cannot be overlooked. The introduction of foreign materials into the eyeball, even those as biocompatible as hydrogels, carries potential risk of immunogenicity, which can lead to inflammation, discomfort, and even rejection. Within this frame, the path forward for hydrogel-based drug delivery systems is paved with both promise and obstacles. Addressing these limitations requires a concerted effort from multidisciplinary teams to refine hydrogel formulations, enhance their interaction with ocular tissue, and tailor their degradation profiles for optimal performance.

## 4. Treatments Based on Hydrogels for AMD

Given the ever-increasing elderly population, AMD treatment is recognized as a significant public health concern. However, the existing curative method is insufficient to cure the disease. Encouragingly, recent developments in the hydrogel-based anti-VEGF delivery system and tissue engineering have demonstrated promising potential in treating AMD.

### 4.1. Hydrogel-Based Anti-VEGF Drug Delivery System

#### 4.1.1. Pre-Clinical Trials

**Figure 3:**
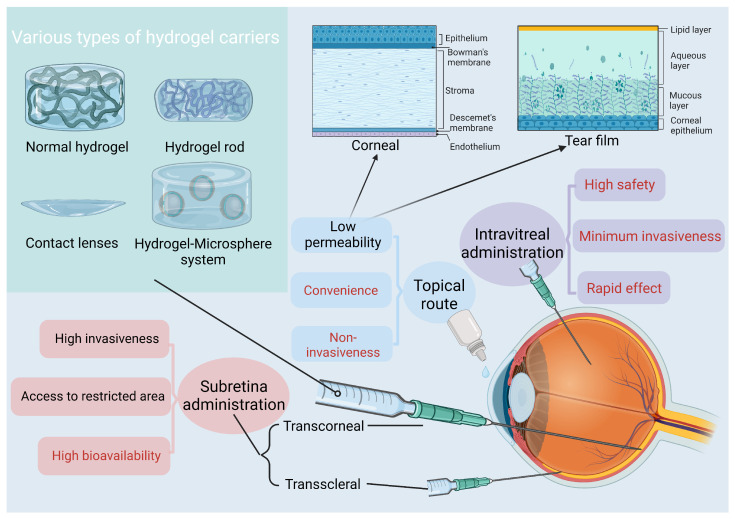
Various ocular injection modalities and their respective merits and demerits. Created with BioRender.com, accessed on 22 January 2024.

In wet AMD, the progressive growth of fragile blood vessels is mainly triggered by the VEGF-related signaling pathway. Hence, anti-VEGF treatment targeting neovascularization holds immense potential in AMD therapy. To date, five available anti-VEGF drugs, namely bevacizumab (Avastin™), ranibizumab (Lucentis™), aflibercept (Eylea™), brolucizumab (Beovu™), and faricimab (Vabysmo™), have been used to inhibit choroidal neovascularization [56,57] (Table 1). Bevacizumab is a full-length VEGF monoclonal antibody that exhibits the capability to counteract all homologous isoforms of VEGF-A. Ranibizumab shares similarity with bevacizumab, as both can bind to homologous isoforms of VEGF-A. However, ranibizumab is composed solely of the antigen-binding fragment derived from bevacizumab. Brolucizumab, a humanized single-chain antibody fragment, is also capable of targeting all subtypes of VEGF-A. It exhibits a superior affinity compared with bevacizumab and ranibizumab. Aflibercept is a human recombinant fusion protein that acts on VEGF-A, VEGF-B, and placental growth factor (PLGF) [58]. Recently, faricimab was approved by FDA as a newly developed inaugural bispecific antibody formulated for intraocular application. It interacts with both VEGF-A and angiopoietin-2 (Ang-2) through independent binding [59]. Although these anti-VEGF drugs have a high degree of specificity and good efficacy, multiple intravitreal administrations catering for their optimal effect will incur potential complications, such as endophthalmitis, intraocular pressure elevation, and rhegmatogenous retinal detachment [6]. Regrettably, alternative methods of administration fail to attain the desired therapeutic efficacy. For instance, the topical delivery of anti-VEGF agents yields inadequate concentrations owing to various biological barriers within the eyeball. The interest in contact lenses loaded with ophthalmic drug has increased, driven by their superior corneal bioavailability, prolonged release course, and improved patient compliance in comparison with topical delivery [60]. However, no pre-clinical or clinical trial employing drug-loaded contact lenses has been identified for treating AMD. Delivery via subretinal methods utilizing transconjunctival or transscleral routes faces challenges due to high invasiveness and limited access to a local region [61]. In contrast to the aforementioned administration methods, intravitreal injection continues to stand as the only competitive method for drug delivery [5] (Figure 3). In order to reach rational therapy strategies, various methods have been explored, including treat-and-extend regimes as a dosing strategy [62] and incorporating photodynamic therapy as an adjunctive treatment. Notably, the hydrogel-based anti-VEGF delivery can reduce the frequency of necessary injections, thereby introducing innovative prospects for AMD treatments.

Hydrogels, as a promising three-dimensional network, boast a large number of characteristics for loading anti-VEGF drugs, including large dug payload, drug protection, biocompatibility, and sustained release. Due to its porous structure, plenty of anti-VEGF drugs can be loaded into a matrix by the absorption of hydrogels. Moreover, their cross-linking structure allows anti-VEGF agents to remain bioactive through preserving the tertiary and quaternary structure from enzymatic reactions. The sustained release of the loaded drug is the main property of hydrogel-based drug delivery systems. There is a burst release phase at the initial stage due to the rapid diffusion of drug molecules into the vitreous before the complete cross-linking of hydrogels. Subsequently, the degradation of the hydrogel mediates the remaining drug release through the breakdown of the cross-linked matrix [5].

**Figure 4:**
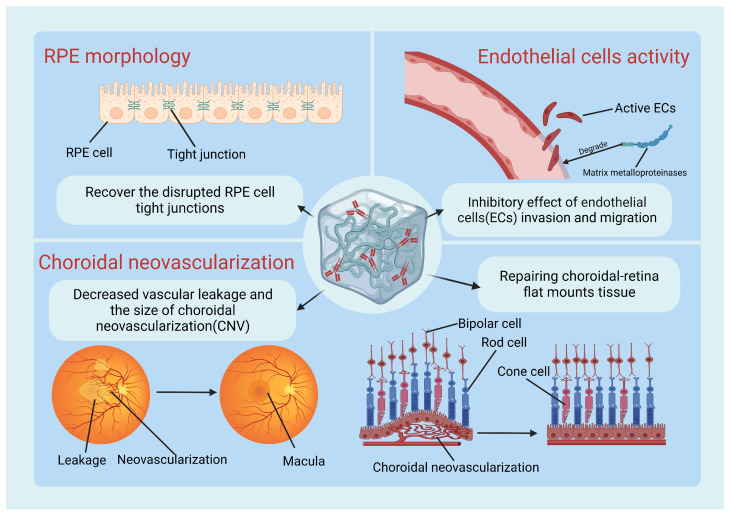
The multifaceted therapeutic effects of hydrogel-based anti-VEGF delivery systems. Created with BioRender.com, accessed on 22 January 2024.

Recently, studies aiming to optimize hydrogel-based anti-VEGF drug delivery systems have achieved a surge of developments (Table 2). They have demonstrated compelling therapeutic efficacy in alleviating symptoms of AMD, including restoring RPE morphology, suppressing endothelial cell apoptosis, and ameliorating choroidal neovascularization (CNV) (Figure 4). Owing to its minimal inflammatory and antigenic properties, hyaluronic acid (HA) acts as a cornerstone in ocular drug delivery, bolstering the biocompatibility and biodegradability of hydrogels. A study delved into the successful development of a sustained anti-VEGF drug delivery system using HA cross-linked with poly (ethylene glycol) diacrylate hydrogels, achieving a remarkable 6-month release period [50]. A similar outcome was achieved by Duan, N., et al. using HA cross-linked with pluronic 127 hydrogels for a prolonged 7-week release [52]. Moreover, hydrogels self-assembling with peptide amphiphile molecules arouse wide interests for their latent potential in ranibizumab delivery. Adjusting the molecule quantity of amphiphile peptide during hydrogel production can easily regulate the released dynamics of the loaded drug. At a ratio of 1:2 between ranibizumab and peptide amphiphile molecule, only 18.8% of the drug was released within 150 h, indicating significant improvement for achieving sustained release [63]. Furthermore, a tetra-armed polyethylene glycol (tetra-PEG) hydrogel has been designed to load bevacizumab. This tetra-PEG hydrogel exhibits high transparency and deformability due to its highly homogenous network. By the second week, the hydrogel system had only gradually released 43.3% of the encapsulated drug, significantly prolonging the efficacy of bevacizumab [64]. In view of their optimal functions, these biomaterials are regarded as excellent candidates for hydrogel-based drug delivery systems, offering viable treatment methods for wet AMD.

A recent study designed the hydrogel rod, a pre-cross-linked rod-like hydrogel delivery system [49]. This hydrogel rod can overcome the limitations such as the dilution of the hydrogel precursor solution with body fluids, the imprecise control over gel formation time, and the g susceptivity to disturbances within the intraocular milieu. During the application of in situ forming hydrogels, the initial burst release poses an unsatisfactory challenge, resulting in a marked loss of anti-VEGF agent. However, the hydrogel rods showcase reduced initial burst release due to pre-cross-linking compared with that of the in situ forming hydrogels. It is promisingly capable of extending the duration of sustained release to 120 days. Furthermore, the hydrogel rod significantly extends the half-life of bevacizumab post-injection when compared with that of in situ forming hydrogels. The half-life is 140 h in the vitreous body and 480 h in the retina for hydrogel rods, whereas for in situ forming hydrogels, it is 68 h in the vitreous body and 121 h in the retina [49]. In another novel drug delivery system, microspheres are inserted into the hydrogel platform to load the anti-VEGF drug. Osswald, C.R., et al. have designed a microsphere–hydrogel delivery system that enables sustained drug release for almost 200 days [65]. In this system, poly (ethylene glycol)-co-(L-lactic acid) (PLGA) microspheres were suspended within a biodegradable poly (ethylene glycol)-co-(L-lactic acid) diacrylate (PEG-PLLA-DA)-N-isopropylacrylamide (NIPAAm) hydrogel. The aflibercept was loaded with PEG-PLLA-DA/NIPAAm hydrogel, which can maintain the therapeutic efficacy for a continuous period of 6 months in treating CNV lesions. In this context, the microsphere–hydrogel delivery system offers advantages over the bimonthly bolus regimen, primarily in terms of reducing the frequency of injections and minimizing the overall dosage. The evidence lies in the contrast between a single injection of 1 μg aflibercept within the microsphere–hydrogel delivery system versus the administration of 200 μg of the drug in each bolus injection. Over a span of 6 months, this accumulation results in a total dosage of 600 μg for the bolus regimen [37]. It has been shown that the microsphere–hydrogel delivery system with 1 × 10^−3^ mol/L cross-linker concentration and less than 20 mg/mL microsphere loadings would be more promising for future application [66].

Neuroinflammation plays a significant role in the pathological process of AMD. Specifically, previous studies have demonstrated that pro-inflammatory cytokines, including interleukin-6 (IL-6) and interleukin-8 (IL-8), typically drive the development and progression of AMD. Furthermore, interleukin-17 (IL-17) can stimulate its receptor on the RPE, triggering an upregulation of antioxidant proteins [67]. Another study revealed that IL-17 can initiate the production of IL-6. This cascade of cytokines orchestrates the metabolic reprogramming of subretinal microglia, thereby initiating neuroinflammation in the degenerative retina [68]. Notably, inflammatory cytokines can also potentiate the secretion of VEGF, which subsequently induces pathological choroidal neovascularization [69]. In a comparative study, researchers found that in the group receiving only anti-VEGF drug injections, its initial two-week efficacy led to a visual improvement, followed by a gradual decline over time. However, in the group receiving an injection of betamethasone phosphate-based hydrogel (BetP-Gel) loaded with an anti-VEGF drug, the improvement of retinal function was notably significant under both photopic and scotopic conditions for four weeks. Mechanistic experiments show that BetP-Gel inhibits the excessive secretion of VEGF by suppressing the TNF/NF-κB/pro-inflammatory cytokine pathway. Likewise, another study found that PEG-PLLA-DA/NIPAAm hydrogels loaded with aflibercept and dexamethasone (a steroid drug) did not notably alter the drug release kinetics in comparison with their individual releases [40]. This strategy of co-loading anti-inflammatory cytokines and anti-VEGF drugs with a hydrogel can minimize the immune response caused by the delivery systems. Simultaneously, it fundamentally suppresses the inflammation-mediated pro-angiogenic effects, curbing the process of neovascularization.

Remarkably, the development of 3D printing offers a novel approach to overcome the challenge of burst release and achieve controlled release of drugs. This innovative technology allows for the choice of various materials with varying degradation rates, effectively regulating the release kinetics of the loaded drug [70]. Moreover, 3D printing technology can provide personalized medical treatment. We can use this technology to design high-resolution structures and geometries with specific amounts of drugs [71], offering a new idea to achieve personalized treatment for AMD. During the process of clinical translation, the hydrogel-based anti-VEGF drug delivery system is confronted with obstacles. Due to their polymer composition and the considerable water content within their structure, hydrogels typically exhibit significant susceptibility to terminal sterilization methods [72]. However, traditional sterilization methods, such as heat, radiation, and gas sterilization, are aggressive enough to affect the properties of some biodegradable hydrogels. On the other hand, novel sterilization techniques, such as ozone and supercritical carbon dioxide sterilization, present extremely low impacts on hydrogel properties, though they may lead to the denaturation of loaded protein drugs [73]. Therefore, great endeavors should be made to ensure both effective sterilization and the preservation of the intended properties of loaded drugs.

#### 4.1.2. Clinical Trials

Axitinib is a small-molecule tyrosine kinase inhibitor that can antagonize VEGF receptors 1, 2, and 3, exhibiting the highest affinity for receptor 2 [58]. OTX-TKI is an investigational biodegradable axitinib-loaded hydrogel implant designed by Ocular Therapeutix™. Its intricate three-dimensional network encapsulates axitinib particles, and upon administration, it undergoes hydration and dissolution, gradually releasing the entrapped drug into the surrounding tissue. Sized comparably to a fine fiber (using a 25–27 g needle), it facilitates sustained release for a period of 6 months or more [58]. Currently, OTX-TKI has been administered intravitreally in individuals diagnosed with wet AMD, and a phase I trial (NCT03630315) is being performed to evaluate the safety, tolerability, and efficacy of OTX-TKI [74]. The trial is actively enrolling and examining groups of participants with varying OTX-TKI doses: cohort 1 (200 µg), cohort 2 (400 µg), cohort 3a (600 µg), and cohort 3b (400 µg + anti-VEGF). The results demonstrate perfect tolerability of OTX-TKI in treating AMD patients. Biological assays detect a reduction in subretinal fluid in the 400 µg and 600 µg dosage groups. An immediate reduction in subretinal fluid was observed in two patients with the combination of OTX-TKI and an anti-VEGF agent. Treatment durability tests revealed effectiveness lasting up to 12 months in the 400 µg dosage group [75]. Complete biodegradation was observed in all patients, with limited and monitorable movement of the implant. No significant adverse event was reported, such as endophthalmitis, retinal detachment, retinal vasculitis, implant migration into the anterior chamber, and elevated intraocular pressure.

Recently, Ocular Therapeutix™ initiated another clinical trial of OTX-TKI in 21 patients with wet AMD, primarily in the United States (NCT04989699). The trial is planned across multiple centers, employing a double-masked, randomized, parallel-group design and aiming to assess the safety, tolerability, and efficacy of OTX-TKI (axitinib implant) [76]. OTX-TKI exhibited the potential to sustain visual acuity and central subfield thickness (CSFT) comparably to aflibercept, accompanied by an 89% decrease in treatment frequency over a 12-month span [77]. It is believed that these trials may introduce an innovative treatment that can profoundly impact individuals contending with wet AMD.

### 4.2. Hydrogel Applications in Tissue Engineering and RPE Transplantation

In the past decades, hydrogel-based transplantation therapies have been developed into a promising treatment for AMD, especially in cases with RPE impairments (Table 3). Therapeutically, cells have mainly been transplanted into the subretinal space by suspension injection [78]. However, this may lead to the maldistribution of non-functional cell clusters and local inflammatory response, which subsequently dampen the cell vitality and implantation success rate. Advantageously, hydrogels with a similar structure and biological properties to the ECM can maintain a stable microenvironment and offer a biodegradable scaffold for encapsulated cells, thereby minimizing immunogenicity during the treatment. The materials used for hydrogel-based transplantation mainly include hyaluronic acid, gellan gum, alginate, chitosan, fibrinogen, poly (ethylene glycol), and methylcellulose. The distinctive biological and mechanical properties inherent to each material can be harnessed for specific applications, but there are several disadvantages when used alone, such as low mechanical strength, poor gelation capacity, limited cell accommodation, compromised cell viability, etc. Hence, it is feasible to create blends that combine or enhance various materials to achieve desired outcomes. For example, a gelatin/gellan gum/glycol chitosan ternary hydrogel was designed in 2020 [79]. This cross-linked structure can enhance the mechanical properties of the hydrogels and provide a conducive microenvironment for the interaction of the cell and matrix.

Recently, gellan gum (GG), a water-soluble anionic polysaccharide mixed with various other organic and inorganic polymers, presents impressive mechanical functionalities, satisfactory biocompatibility, minimal cytotoxicity, etc. Brzozowski, P., et al. showed that a GG/HA hydrogel promoted ARPE-19 cell growth, viability, and adhesion in vitro with fast stress relaxation, which is considered an important mechanical cue for the control of cellular activities [80]. In this study, GG was doped with divalent (Ca^2+^) cations to induce ionic cross-linking [96]. It has been reported that such structure provides a stable support for cell viability and proliferation [97]. In another in vitro study, researchers established a PEG/GG hydrogel to overcome the limitation of the fragility of GG scaffolds during loading in ARPE-19 cells. The PEG/GG hydrogels exhibited superior biocompatibility, enhanced cell adhesion, and promoted cell growth. This system could also increase the expression of RPE-specific genes, such as RPE 65 (an isomerase enzyme in the visual cycle) and cellular retinaldehyde-binding protein (CRALBP), making it a potential candidate for a hydrogel-based transplantation material for AMD [90].

RPE is a kind of simple cuboidal epithelium whose quiescent monolayer structure is crucial for functions pertaining to phagocytosis of the distal segments of the photoreceptor, light energy absorption to optimize vision, and blood retinal barrier development. In several hydrogel-based transplantation clinical trials, parylene and polyester are utilized to preserve the integrity of RPE [98,99]. Nonetheless, animal studies show that the material persisted between the RPE and choroid, leading to fibrosis and localized inflammation post-implantation. To solve this problem, fibrin hydrogel is utilized as a supporting scaffold for RPE cells adhesion. This scaffold facilities the formation of a functional monolayer structure within 2 weeks in the eyes of mice [85] and 8 weeks in female pigs [84]. Fibrin is a cross-linking fibrillar network formed after the spontaneous activation of fibrinogen in the organism. The autologous fibrin hydrogels can be degraded by RPE cells. Therefore, hydrogels are loaded with the protease inhibitor aprotinin and the added fibrinolytic enzymes rapidly degrade the fibrin support, leaving an intact RPE monolayer [83]. Wei, Y., et al. demonstrated that human embryonic stem cell-derived retinal pigment epithelium (hESC-RPE) cells transported through the fibrin hydrogel retained the monolayers with a cobblestone appearance, polarized morphology, specific protein expression, and maturation-related secretion capability in vivo [85].

Emerging studies show that bioactive molecules such as taurine, curcumin, dopamine, and synthetic peptides can improve the functional behaviors of the retina and promote the formation of RPE monolayers. For instance, taurine plays an important role in the normal function of synaptic connections, antioxidant protection, and RPE phagocytosis [100]. Shin, E.Y., et al. designed a taurine-loaded alginate hydrogel to encapsulate rabbit RPE cells. It was injected into the subretinal space, and the researchers found that this hydrogel was able to drive the expression of RPE-specific genes and retinal regeneration [81]. Curcumin is a notable bioactive molecule that can enhance the therapeutic properties of alginate hydrogels. A previous study showed that curcumin/alginate hydrogel presents excellent biocompatibility and increased ECM formation. Specifically, this hydrogel exhibits a notable 28% increase in proliferation compared to the pure alginate and promotes the expression of essential genes related to retinal functions and matrix production [86]. Furthermore, dopamine, a kind of catechol, has been demonstrated to significantly enhance the performance of hydrogels in transporting cells. The voluminous nature of dopamine was theorized to perturb the molecular conformation of GG’s helix structure, thereby diminishing its physical entanglement. This attribute heightened degradability according to high soluble fraction while concurrently enhancing the desired rheological and mechanical characteristics based on a low compression modulus. In addition, the dopamine-modified hydrogel can not only enhance cell adhesion and migration but also sustain long-term regeneration effects by improving retinal progenitor cell (RPC) synapse formation [82,93]. Through activating the integrin α5β1–PI3K signaling pathway, this hydrogel enhances RPC attachment and facilitates RPC differentiation into photoreceptors. Arg-Gly-Asp (RGD), a synthetic peptide, offers significant advantages over native matrix proteins by acting as a potential ligand for various integrin receptors expressed in the RPE, thereby enhancing retinal regeneration [88,89,101]. In summary, bioactive molecules significantly enhance cell viability, addressing the issue of poor cell survival both in vitro and in vivo within the field of hydrogel engineering.

AMD pathology is characterized by the aberrant interplay between the RPE and photoreceptors. In this context, researchers have launched the hydrogel-based co-transplantation of RPE and photoreceptors [87]. In this study, the hydrogels were used to encapsulate the rod photoreceptor and ARPE-19 cells and then were injected into the subretinal space of a sodium iodate (NaIO_3_)-induced AMD mice model. When transplanted together, both the RPE and photoreceptors exhibited significantly improved survival rates compared with those in their respective single-cell-type controls. Although the underlying mechanism remains elusive, it seems that donor photoreceptors are able to facilitate the survival of the transplanted RPE, consequently enhancing scotopic vision by supporting both donor and host photoreceptors. Notably, photoreceptor loss in AMD patients initiates a constellation of pathological events, termed retinal remodeling, that corrupts retinal integrality and visual circuitry. Retinal remodeling can lead to the deafferentation of the neural retina, retina ganglion cell (RGC) axonal strangulation by retinal vessels, RGC death, and the revision of retinal synaptic connections [102]. Fundamentally, hydrogel-based transplantation therapies are designed on the prerequisite that the downstream neural retinal circuits are normal and intact [103]. Hence, advocating for the implementation of hydrogel-based transplantation therapies in the early stages of AMD, before the remodeling cascades into the inner retina, seems to be judicious.

## 5. Future Perspectives of Hydrogel-Based Treatments

As the frontline treatment for AMD, the intraocular delivery of anti-VEGF agents has encountered complex hurdles. However, hydrogel-based treatments have demonstrated potent therapeutic efficacy and satisfactory safety in clinical work. They substantially reduce the frequency of injections and the burden of patients while enhancing drug stability, biocompatibility, release duration, and biodegradability to fulfill the unmet need. Moreover, the utilization of hydrogels in tissue engineering seeks to elevate the vitality of RPE cells and strengthen their ability to form functional monolayers, thereby instilling hope for vision restoration in these AMD patients. Therefore, the integration of hydrogel-based therapies into clinical research represents a notable advancement, signifying a substantial leap in leveraging hydrogels’ potential within ophthalmology practice. Aiming to drive the broader and deeper application of hydrogel, we aspire to present some advancements to facilitate their translation into clinical applications. Subsequent research endeavors should concentrate on safety assessments and the refinement of these promising delivery systems. Moreover, the rapid development of bioengineering methods such as 3D printing technology allows for the design of hydrogel structures with specific amounts of drugs and accurate release profiles, introducing a novel concept to tailor treatments for AMD. Furthermore, amidst the rise of gene-editing technologies, their integration with current hydrogel research is poised to achieve impactful therapeutic effects, even paving the way for curing AMD. To sum up, hydrogel-based delivery systems act as promising candidates with admirable traits for solving unmet needs in AMD treatment.

## Figures and Tables

**Table 1:** Available anti-VEGF drugs for AMD treatments [56,57].

Drug	Date of FDA Approval	Type of Molecule	Molecular Weight (kDa)	Standard Dose
Ranibizumab (Lucentis^TM^)	2006	Humanized monoclonal antibody fragment inhibitor of all isoforms of VEGF-A	48	0.5 mg/50 μL
Aflibercept (Eylea^TM^)	2011	Recombinant fusion protein of VEGF receptors 1 and 2	115	2 mg/50 μL
Brolucizumab (Beovu^TM^)	2019	Humanized single-chain antibody fragment inhibitor of all isoforms of VEGF-A	26	1.0 or 6.0 mg/eye
Bevacizumab (Avastin^TM^)	off-label	Humanized monoclonal antibody that targets all isoforms of VEGF-A	149	1.25 mg/50 μL
Faricimab (Vabysmo^TM^)	2022	Humanized bispecific immunoglobulin G monoclonal antibody inhibitor of VEGF-A and Ang-2	150	6 mg/50 μL

**Table 2:** Novel studies of hydrogel-based anti-VEGF agents delivery systems.

Type of Hydrogel	Bioactive Agents	Route	Experimental Models	Sustained Release	Bioactivity Assay	Biocompatibility	Ref.
Poly (ethylene glycol)-co-(L-lactic-acid diacrylate/N-isopropylacrylamide (PEG-PLLA-DA/NIPAAm) hydrogel	Aflibercept and dexamethasone	In vitro	In vitro	224 days	ELISA and cell proliferation assay	-	[40]
Super-molecular nanofiber hydrogel	Betamethasone phosphate and an unspecified anti-VEGF drug	Intravitreal injection	Rats	At least 4 weeks	CCK-8, Transwell, EdU, and live/dead assays	Proven on histological examination	[51]
Hyaluronic acid (HA) /poly (ethylene glycol) diacrylate hydrogel	Bevacizumab	Suprachoroidal administration	Rabbits	At least 6 months	ELISA assay	Proven on histological examination	[50]
Tetra-armed polyethylene glycol (tetra-PEG) hydrogel	Bevacizumab	In vitro	In vitro	At least 2 weeks	ELISA, HPLC, and VEGF bioassay kit assay	-	[64]
Gelatin/PEG/tyramine (GPT) hydrogel	Bevacizumab	Intravitreal injection	Rabbits	At least 4 months	Cell proliferation assay	Proven on rabbit TNF-α DuoSet ELISA kit assay	[49]
Hyaluronic acid (HA)/pluronic 127 hydrogel	Ranibizumab	Intravitreal injection	Rabbits	At least 7 weeks	CCK-8 assay	Proven on histological examination	[52]
Peptide amphiphile hydrogel	Ranibizumab	Intravitreal injection	Rabbits	At least 7 days	MTT assay	-	[63]
Poly (lactic-co-glycolic acid) (PLGA) microsphere within PEG-PLLA-DA/NIPAAm hydrogel	Aflibercept	Intravitreal injection	Rats	At least 6 months	H&E staining assay	Proven on histological examination	[36]

**Table 3:** Novel studies on hydrogel-based transplantation for the treatment of AMD.

Type of Hydrogel	Retinal Cell Type	Cellular Compatibility	Experimental Models	Biocompatibility	Ref.
Gellan gum/hyaluronic acid hydrogels	ARPE-19	Live/dead assay	In vitro	In vitro	[80]
Taurine-loaded alginate hydrogels	Rabbit RPE	Live/dead assay and MTT	Nude mice	Immunohistochemistry analysis	[81]
Dopamine-functionalized gellan gum hydrogels	ARPE-19	Live/dead staining and gene expression analysis	In vitro	In vitro	[82]
Fibrin hydrogels	iPSC-RPE cells	Live/dead assay and gene expression analysis	In vitro	In vitro	[83]
Fibrin hydrogels	Non-cells	-	Female pigs	H&E staining	[84]
Fibrin hydrogels	ARPE-19 and hESC-RPE cells	Live/dead assay	C57BL/6J mice	H&E staining	[85]
Curcumin/alginate hydrogels	Rabbit RPE	Live/dead assay, MTT analysis, and gene expression analysis	In vitro	In vitro	[86]
Hyaluronic acid/methylcellulose hydrogels	Rod photoreceptor and ARPE-19	-	C57Bl/6J mice	Immunohistochemistry analysis	[87]
Arg-Gly-Asp/alginate hydrogels	hESC-RPE cells	-	RCS rats	H&E staining	[88]
Arg-Gly-Asp/alginate hydrogels	hESCs/hiPSCs embryoid bodies	Gene expression analysis	In vitro	In vitro	[89]
Polyethylene glycol/gellan gum hydrogels	ARPE-19	MTT and gene expression analysis	In vitro	In vitro	[90]
Gelatin/gellan gum/glycol chitosan ternary hydrogels	ARPE-19	Live/dead assay, MTT analysis, and gene expression analysis	In vitro	In vitro	[79]
Gelatin/hydroxyphenyl propionic acid hydrogels	Retinal progenitor cells (RPCs)	EthD-1 staining	Female Long Evans rats	Immunohistochemistry analysis	[91]
Chitosan hydrochloride/oxidized dextran hydrogels	Retinal progenitor cells (RPCs)	Live/dead assay and CCK-8 analysis	In vitro	In vitro	[92]
Gelatin/hyaluronic/polydopamine hydrogels	Retinal progenitor cells (RPCs)	Live/dead assay and inflammatory and apoptotic factor expression levels	Nude mice	H&E staining	[93]
IGF-1 loaded Src homology3-binding peptides hyaluronan/methylcellulose (HAMC) hydrogels	hESCs	Immunostaining	In vitro	In vitro	[94]
Glycol chitosan coated cerium oxide nanoparticles with alginate/gelatin hydrogels	ARPE-19	Gene expression analysis	Light-induced Nrf2^−/−^ mice	H&E staining	[95]

## Data Availability

Not applicable.
